# Improvement of germination rate and hybridization to facilitate breeding of an industrial oil crop, *Euphorbia lagascae* Spreng

**DOI:** 10.1186/s13007-024-01141-2

**Published:** 2024-01-24

**Authors:** Maram Istaitieh, Jim F. Todd, Rene C. Van Acker, Mohsen Yoosefzadeh-Najafabadi, Istvan Rajcan

**Affiliations:** 1 Department of Plant Agriculture, University of Guelph, Guelph, ON N1G 2W1 Canada; 2 Ontario Ministry of Agriculture, Food and Rural Affairs, Simcoe, ON N3Y 4N5 Canada

**Keywords:** Flower morphology, Growing conditions, Hybridization, Industrial crops, Plant breeding, Vernolic acid

## Abstract

**Background:**

The potential of plant-based sources of vernolic acid to provide agricultural producers with a market diversification opportunity and industrial manufacturers with a renewable, environmentally friendly chemical feedstock is immense. The herbaceous wild spurge or caper spurge (*Euphorbia lagascae* Spreng) is the most promising source of vernolic acid, containing an average oil content of 50%, of which around 60% is vernolic acid. Its seed yield ranges between 500 and 2000 kg ha^−1^, and a theoretical yield of 180 kg ha^−1^ of pure vernolic acid is possible. The objective of this research was to characterize the flower and whole plant morphology so to allow for the development of a method to efficiently hybridize *E. lagasce* plants for breeding purposes.

**Results:**

In this study, we have characterized the flower and whole plant morphology in detail, thereby, developing an efficient method for hybridization of *E. lagasce* to allow for its breeding and improvement as a novel oil crop. Such method was not described previously in the literature making it difficult to breed this crop. We believe that the method will be of great value to plant breeders working on optimizing the crop, particularly in terms of the development of non-shattering cultivars with enhanced germination potential.

**Conclusions:**

The successful development of this crop through plant breeding could provide substantial economic benefits to farmers by offering them a new industrial oilseed crop. This research could prove invaluable in unlocking the potential of *E. lagasce*, and in turn, the potential of vernolic acid as a renewable, environmentally friendly source of chemical feedstock.

**Supplementary Information:**

The online version contains supplementary material available at 10.1186/s13007-024-01141-2.

## Background

The genus Euphorbia, with over 3000 species, is an abundant source of renewable industrial raw plant-based feedstock [1]. Of particular interest is the species *Euphorbia lagascae* Spreng, commonly known as mole-plant, herbaceous wild spurge, or caper spurge. This species has the remarkable ability to repel moles [2, 3], and is often mistaken for the closely related *Euphorbia lathyris* [4]. Although generally considered to be an annual crop, reports suggest that *E. lagascae* may also be grown as a biennial or perennial plant [2, 3]. This species is native to arid regions of south-eastern Spain and is widely distributed in large areas of Sardinia [2, 4]. Wild accessions of *E. lagascae* grow around cities and along road boundaries when the soil contains high levels of nitrogen [2, 5, 6]. Several studies have revealed the potential of *E. lagascae* as a novel oilseed crop; however, agronomic improvement is necessary to reduce the seed-shattering phenotype [7]. In North Dakota, seed yield ranged from 100 to 200 kg.ha^−1^, largely owing to capsule shattering and resulting seed losses of up to 400 kg.ha^−1^ [4]. To this end, breeding efforts have been undertaken in Spain, Germany, and the Netherlands [2, 8]. Moreover, research on the utilization and cropping of *E. lagascae* in Hungary have highlighted the importance of breeding wild-type genotypes to produce superior agronomic qualities [2, 3].

Recently, global attention has been drawn to *E. lagascae* as a potential oilseed crop due to its high concentration of vernolic acid, an essential epoxy fatty acid used as plasticizers and in other industrial products [4]. Herbicide tolerance studies on non-shattering mutant genotypes in Oregon indicated that *E. lagascae* Spreng had good tolerance to a range of herbicides and the potential to become an important crop and a source of epoxy fatty acids [9]. Additional breeding efforts are needed, however, to further reduce seed shatter and thus improve seed yield [10–12]. Field trials conducted in southern Ontario, Canada [13, 14] revealed that mutant genotypes of *E. lagascae* Spreng were able to thrive under the Ontario weather conditions and yielded 500–2000 kg.ha^−1^. The wild-type plants studied in these experiments had capsule walls consisting of three pericarp layers and the tension within the capsules was generated from the mid-layer (mesocarp) [11]. As the capsules matured and dried, the increase in pressure between the capsule layers caused the capsules to shatter [11]. Mutant genotypes, however, featured lighter, thinner capsule walls and/or the absence of the mesocarp layer; these characteristics resulted in a non-shattering phenotype [2, 3, 11].

In terms of other high vernolic acid plant species, the potential of *Centrapalus pauciflorus* as an industrial oilseed crop with a high level of vernolic acid has been examined, and studies have shown a higher resistance to weeds such as redroot pigweed (*Amaranthus retroflexus*) when compared to traditional oilseed crops such as soybean [15]. This makes *C. pauciflorus* a strong candidate for consideration as a potential oilseed plant in the future [15]. In another study conducted by H Shimelis, PW Mashela and A Hugo [16], field evaluations of 36 accessions of Vernonia (Vernonia galamensis) in 2005 and 2006 indicated significant differences in the content of seed oil, vernolic acid, linoleic acid, oleic acid, palmitic acid and stearic acid, with particularly high levels of vernolic acid in two accessions (Vge-4 and Vge-31) and high seed yields in three (Vge-17, Vge-18 and Vge-19). Therefore, Vernonia can be viewed as an additional source of vernolic acid, albeit at a lower concentration than that found in *E. lagascae* Spreng.

The large-scale production of *E. lagascae* Spreng is hindered by a severe and pervasive seed shattering, rendering it a challenge to breed and domesticate. To this end, a study was conducted to induce genetically stable Ethyl methane sulfonate (EMS) mutated genotypes [12]. The findings of the study revealed that the extent of the non-shattering phenotype was greatly impacted by the environment, with maximum seed yields achieved under stressful conditions such as drought. Additionally, two types of indehiscent phenotypes were identified: those associated with sterility, unaltered by the environment, and those in which a portion of the capsules present an indehiscent phenotype but with no sterility issues per se. In this latter case, any sterility would be the result of different stresses associated with environmental conditions [11]. Thus, breeding to improve field germination and seed retention will lead to an increase in seed yields. The main objective of this study was to characterize in detail the flower morphology and to develop and describe a method for efficient hybridization of *E. lagasce* flowers to enhance the breeding and improvement of this species as an industrial crop. In addition, the latter will provide a mechanism to design crosses between the non-shattering mutants that have germination issues with shattering wild type plants, which have good germination, with the goal of producing non-shattering, good germination cultivars.

## Results

**Fig. 1:**
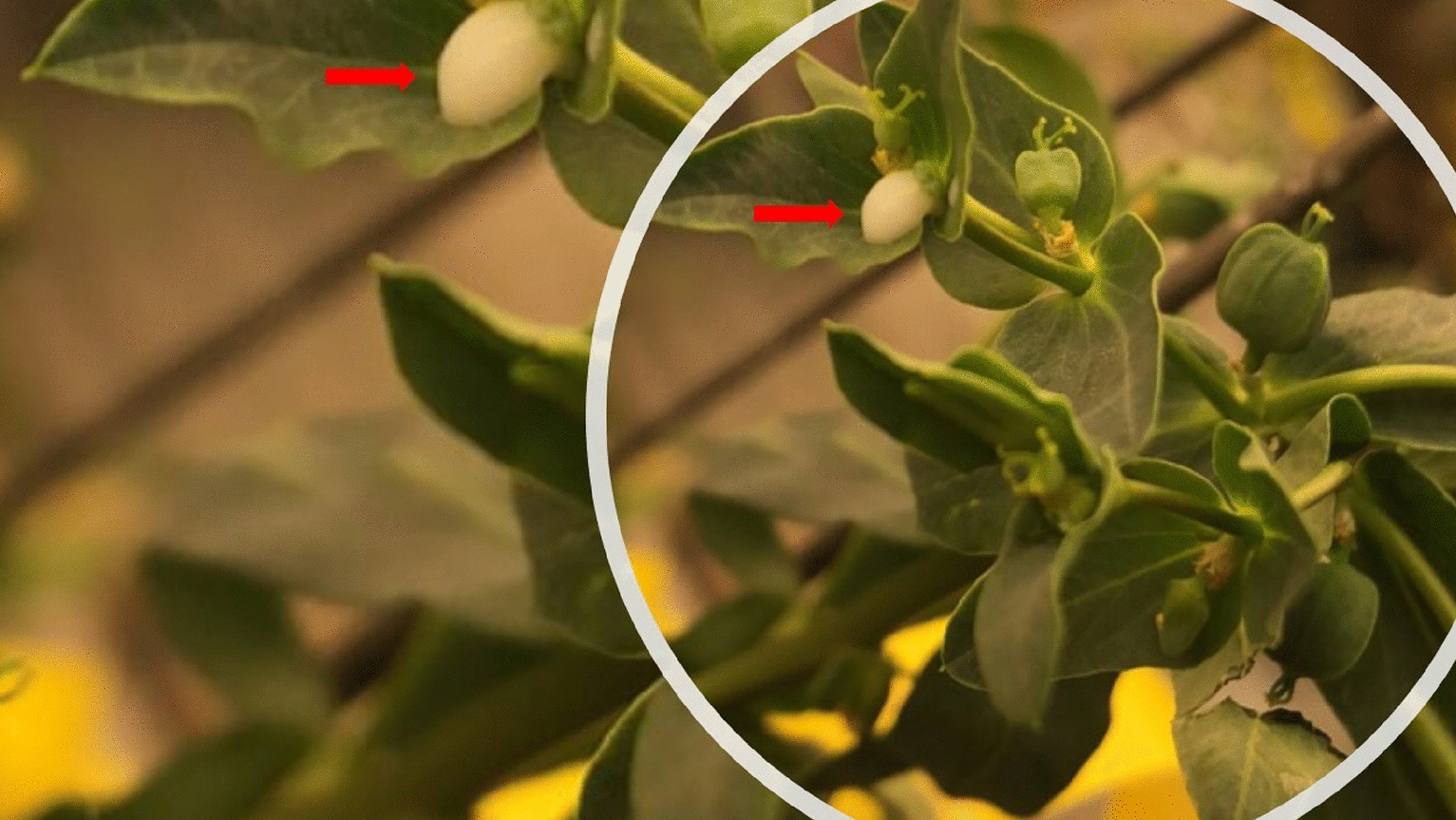
The milky white latex. Red arrows indicate the milky white latex

**Fig. 2:**
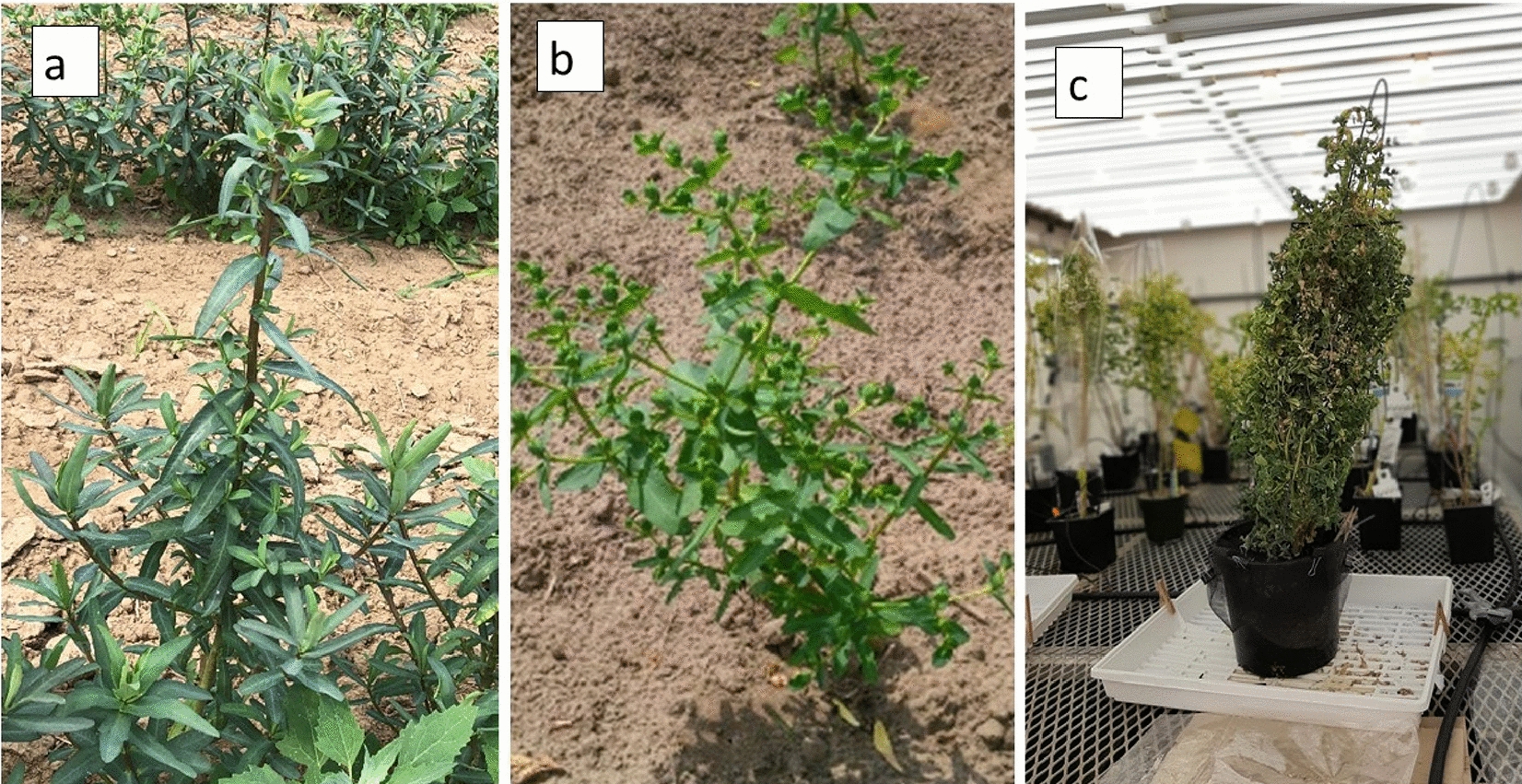
Leaf colours of various genotypes: **a** dark green for mutant, **b** light green for wild- and **c** mix of dark and light green for the hybrid (WT × mutant)

**Fig. 3:**
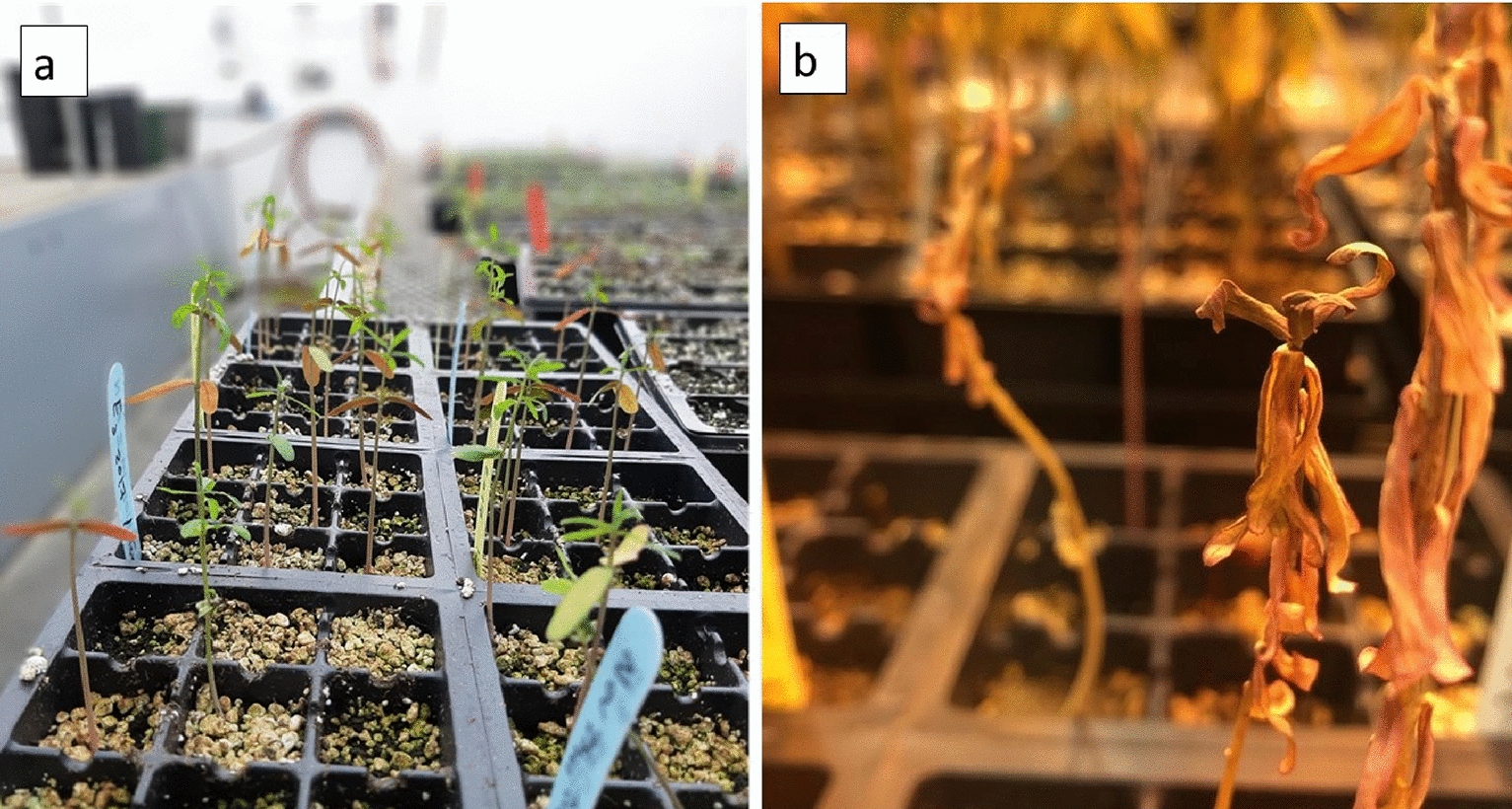
Leaf colour indicating; **a** plants requiring transplanting when the leaves turned red, **b** exposure to hot and dry conditions when the leaves turned yellow then brown

The leaves of the developed *E. lagascae* Spreng populations were lanceolate with a length ranging from 1 to 5 cm (excluding petioles) and producing milky latex (Fig. 1). Differences in some morphological traits, such as leaf colouration, were observed between wild type (WT) and mutant genotypes, ranging from light to dark green (Fig. 2a–c). Furthermore, the leaves of all F_2_ populations turned red due to transplantation stresses, (Fig. 3a) however, transplanted plants turned green and healthy after repotting into 4.5 L containers.

**Fig. 4:**
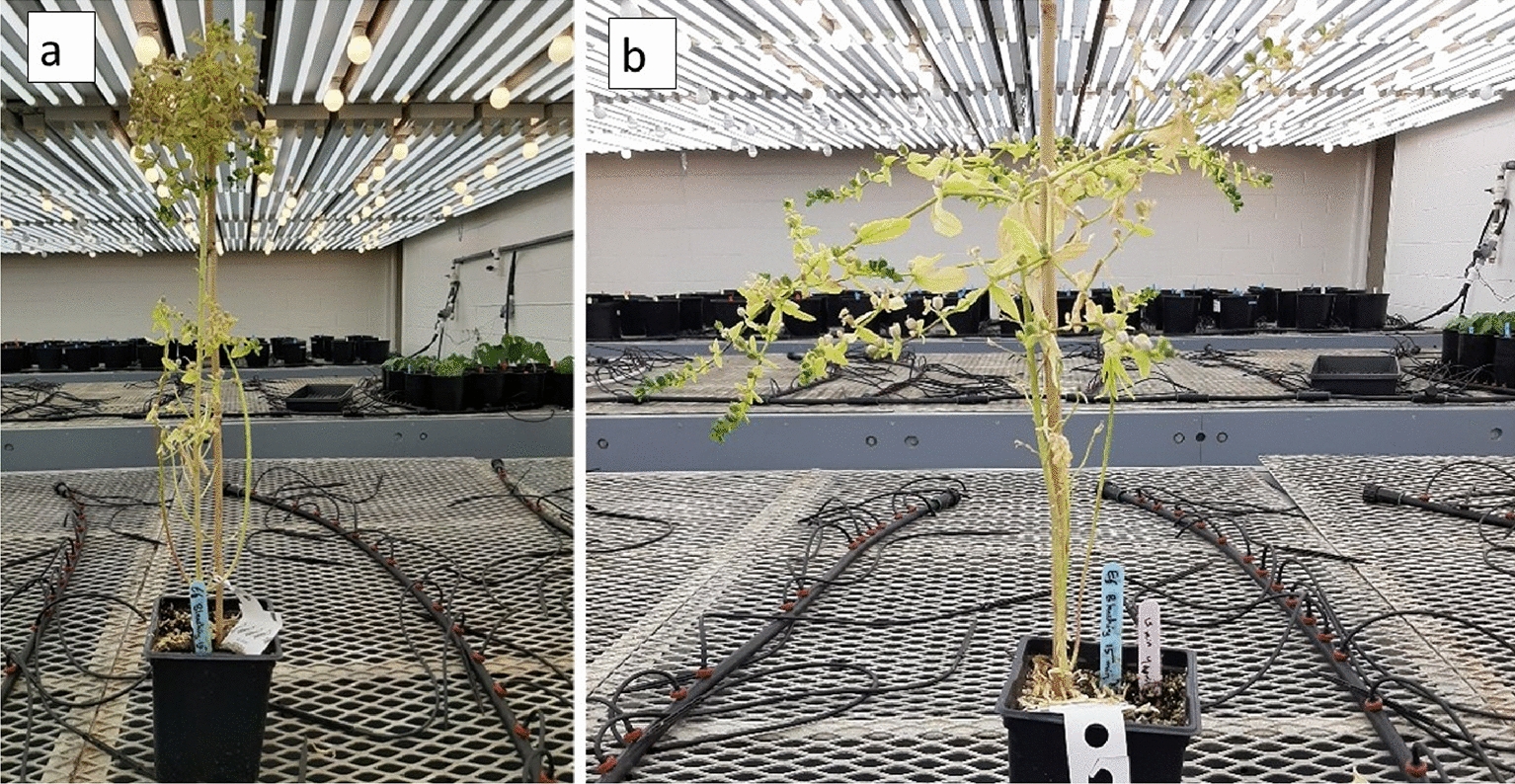
*Euphorbia lagascae* Spreng plants with **a** vertical stems, and **b** horizontal stems

**Fig. 5:**
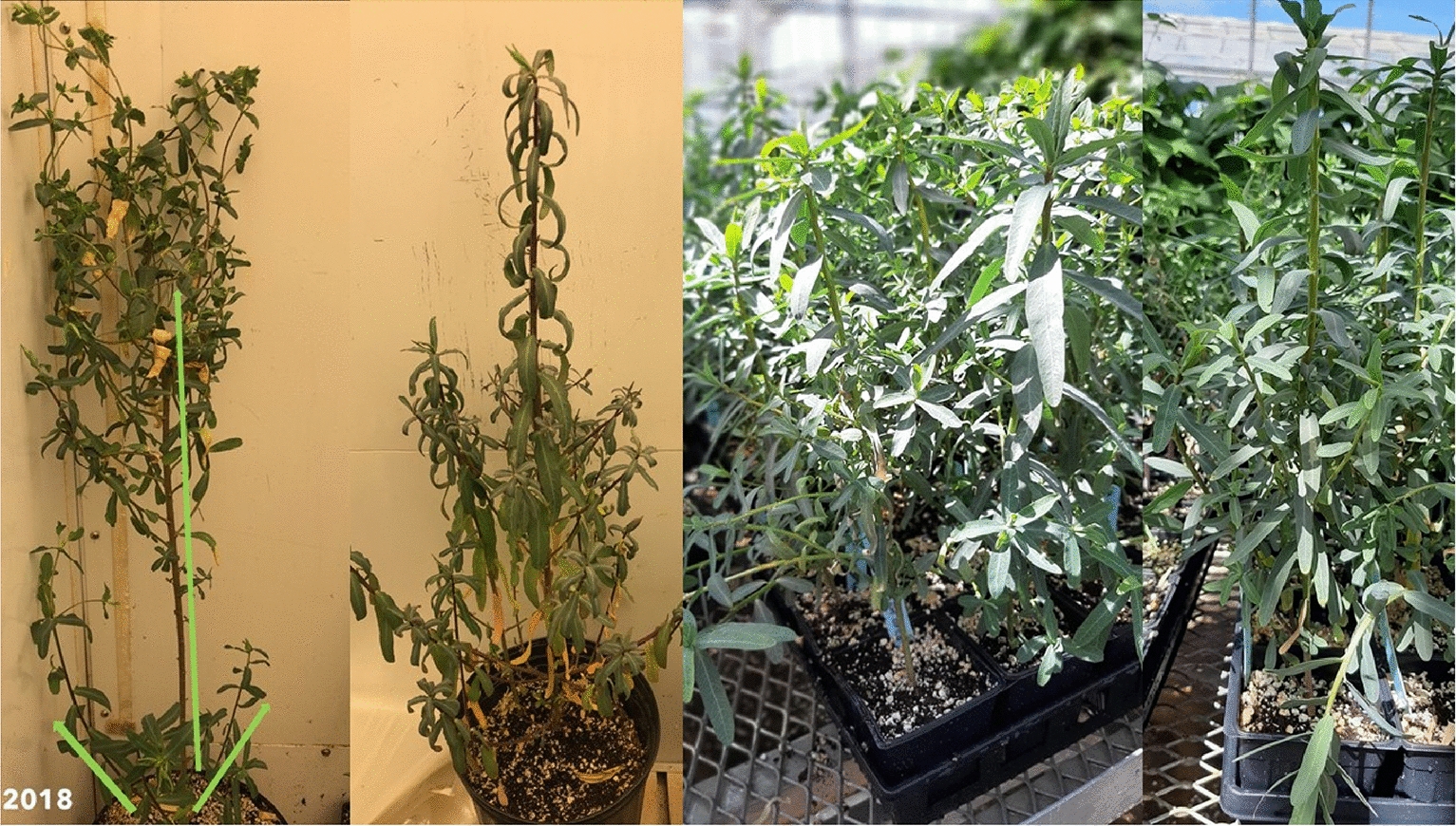
Mutant genotypes with single main erect stem and two crown lateral branches

**Fig. 6:**
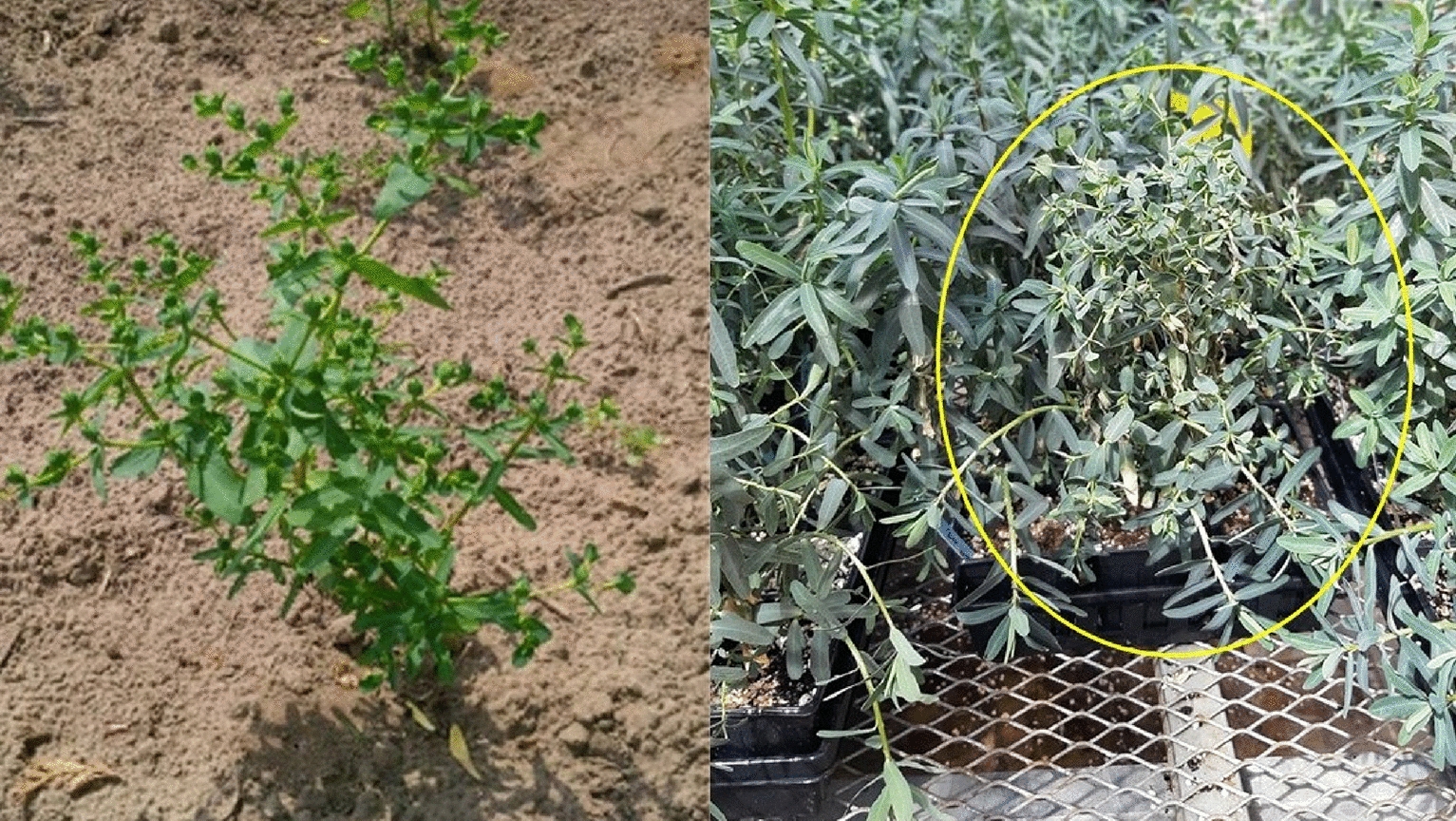
Wild-type genotype with branches cover the main stem

**Fig. 7:**
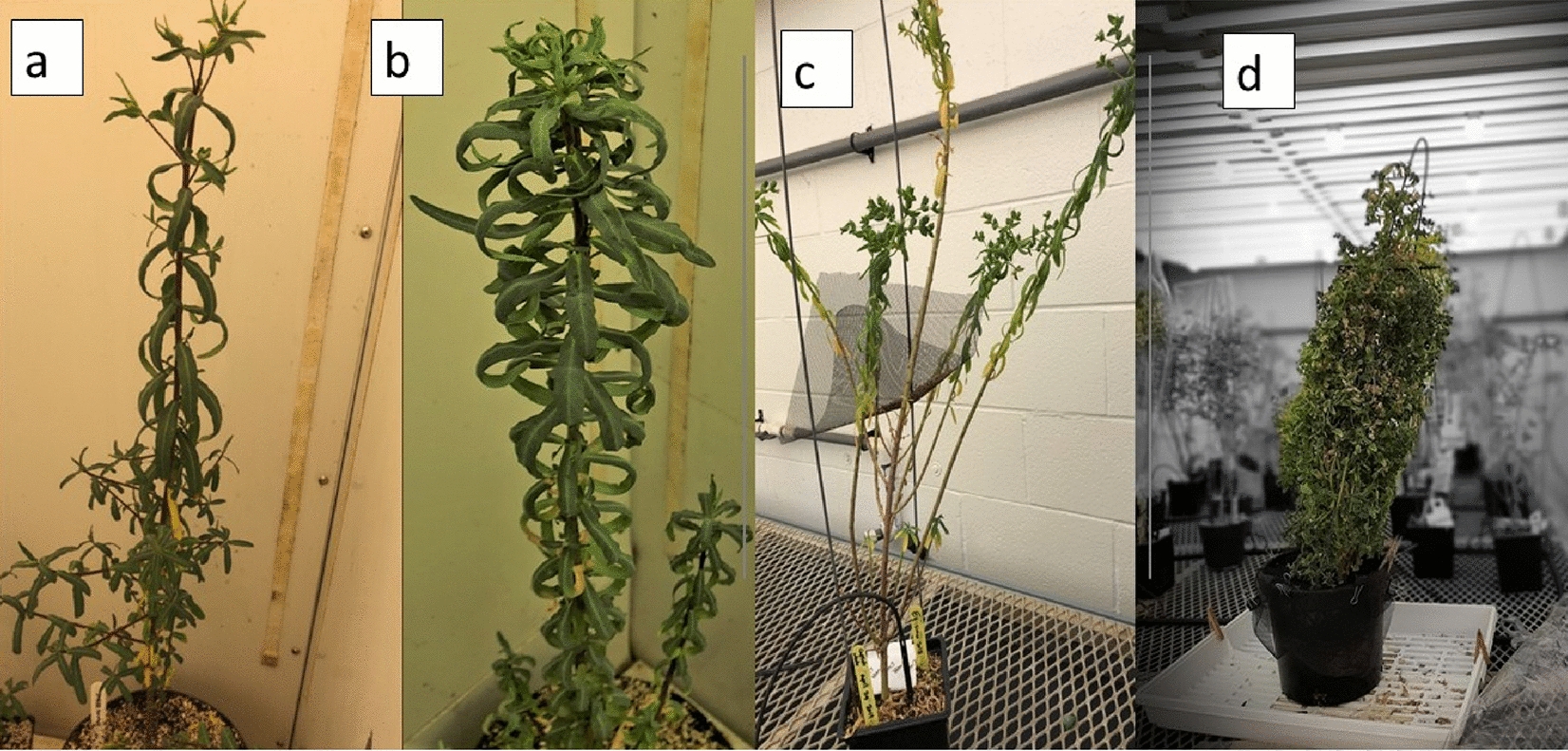
Stem branch types **a** single branch in WT parent, **b** double branches in WT parent, **c** triple branches in WT parent, and **d** hybrid with bunched branches

The stem height of *E. lagascae* Spreng plants ranged from 20 to 80 cm and displayed either a vertical or horizontal phenotype (Fig. 4). All plants with a single main erect stem with two branches arising from crown area were typically more prominent in the mutant genotype (Fig. 5). The WT genotype had many branches covering the main stem, which made distinguishing between the branches very difficult (Fig. 6). The stems of hybrid plants continued to produce new bunched branches indeterminately making the hybrid plants much larger and bushier than the parental lines which typically had single, double or triple branched stems (Fig. 7a–d). However, for all genotypes, removing the apical meristem stimulated the growth of lower branches as plants grew taller.

**Fig. 8:**
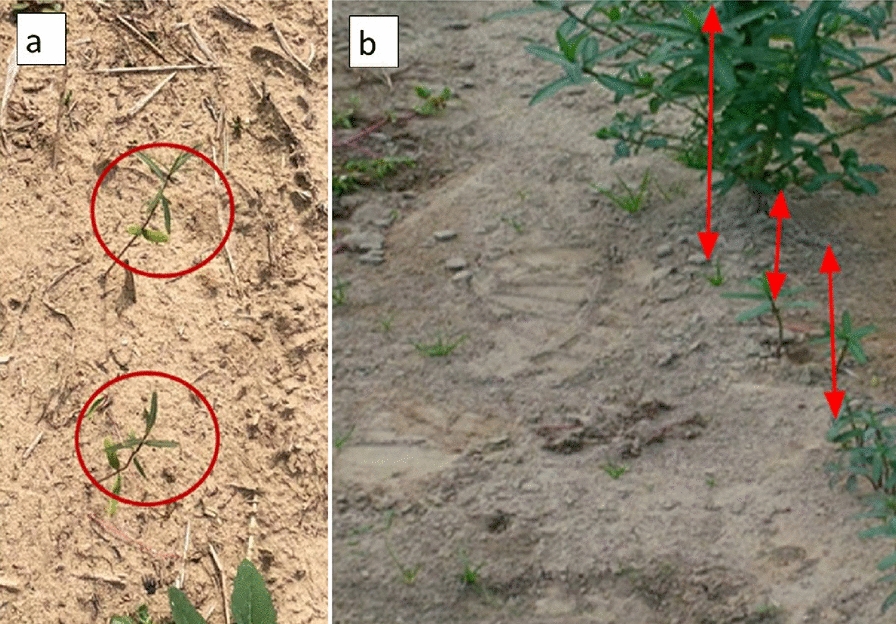
Plants grown directly from seed in the field at the Simcoe Horticultural Experiment Station. **a** Weak thread-like stem, and **b** irregular and low rates of germination

In our study, WT plants grown in the greenhouse frequently exhibited lodging, which was attributed to a combination of genetic and environmental factors; a 10 **℃** increase in temperature resulted in a significantly greater degree of lodging. Concurrently, the mutant genotype EU006 produced thin, weak stems that grew irregularly even when sown at the same time (Fig. 8). This indicates that the lodging phenomenon is not only associated with environmental conditions but is also heavily influenced by genetic components. In terms of root growth and biology, roots penetrate deeply into the soil in the 1st weeks of planting. Under wet and cool soil conditions but *E. lagascae* is susceptible to root rot when grown under Ontario conditions. Under controlled environment conditions, therefore, it is important to keep the soil moist but not overly wet because wet conditions encourage pathogens including rust, fungal gnats, powdery mildew and root rot (*Pythium, Rhizoctonia* and *Fusarium* ssp.).

**Fig. 9:**
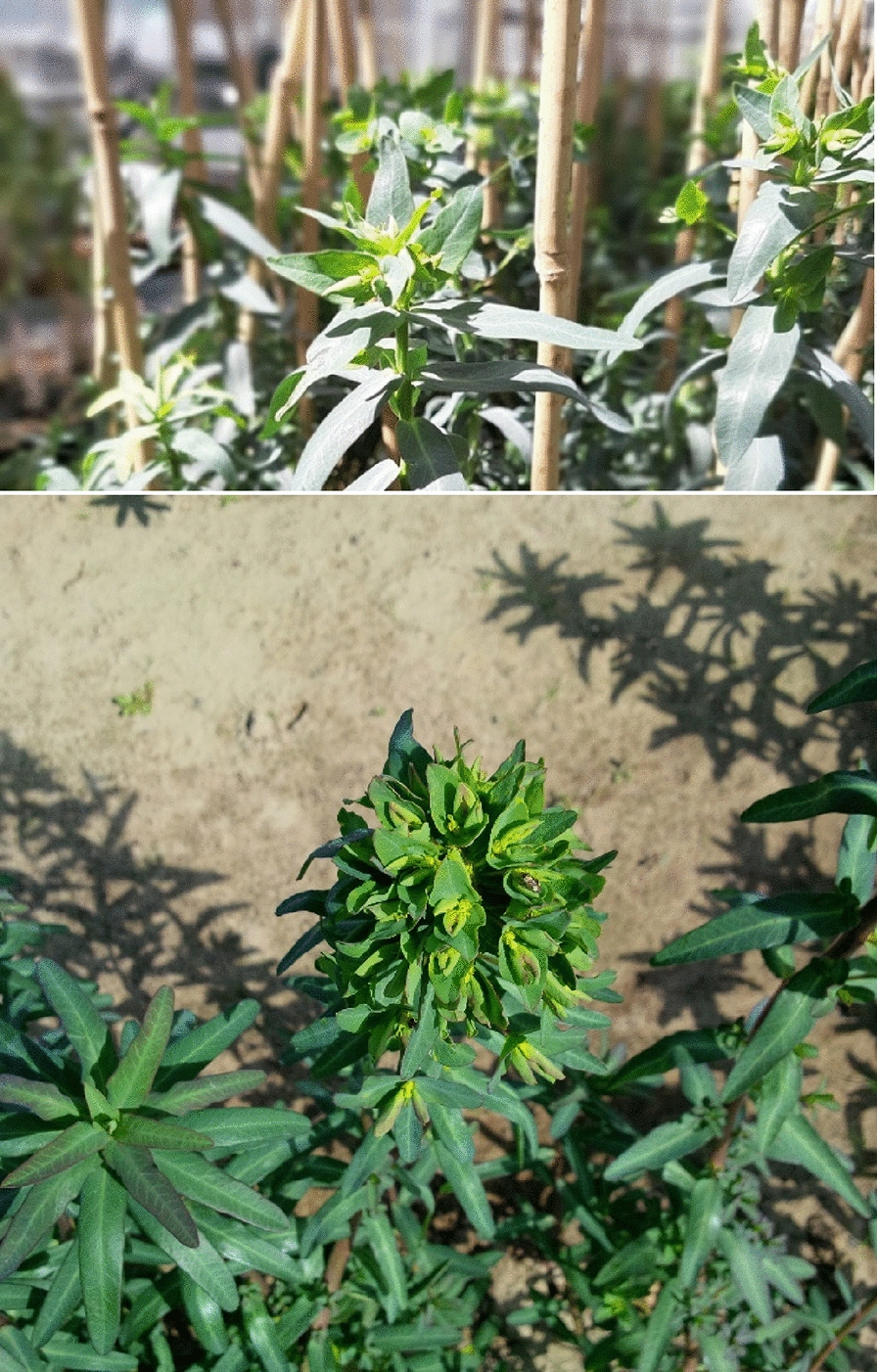
Images of Cyathia with a single female flower (top) and several male flowers (bottom) in one structure (Cyathium)

**Fig. 10:**
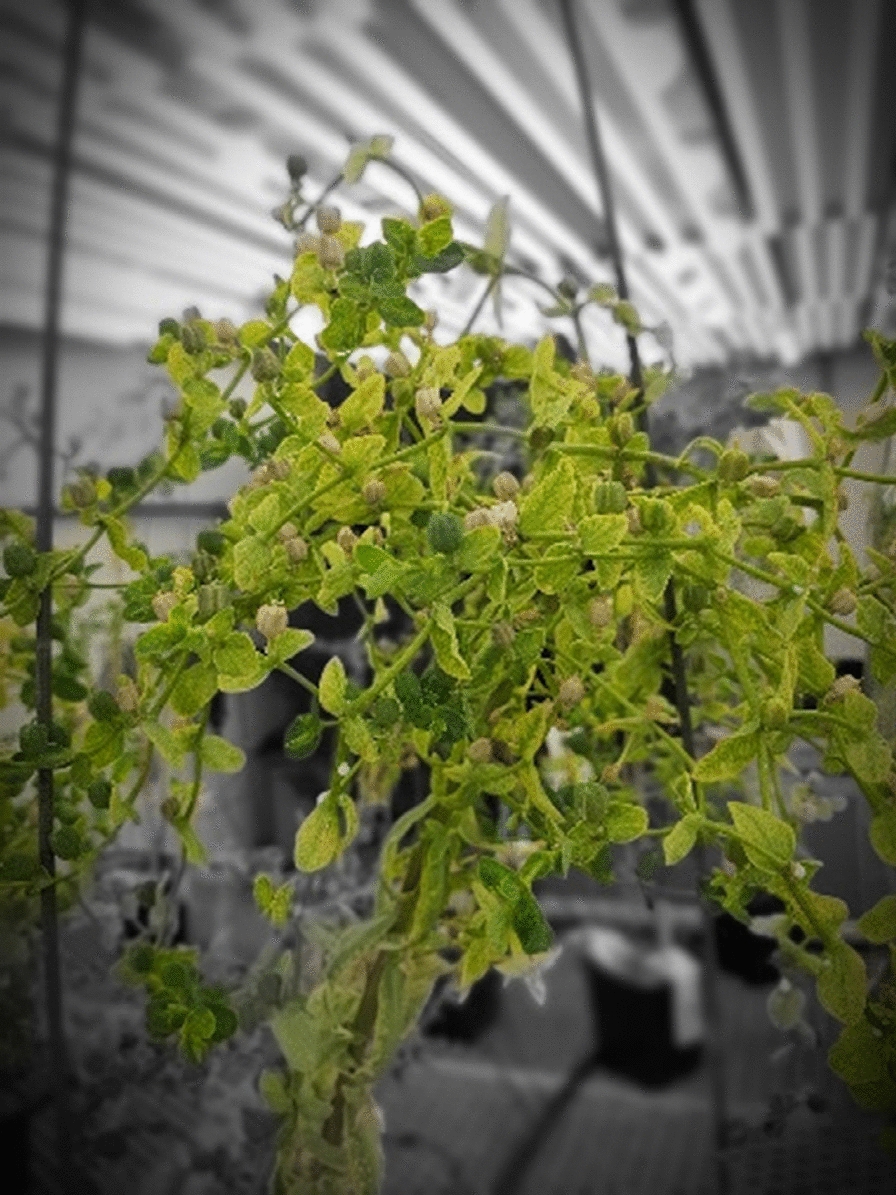
Non-shattering (indehiscent) phenotype of the capsules

**Fig. 11:**
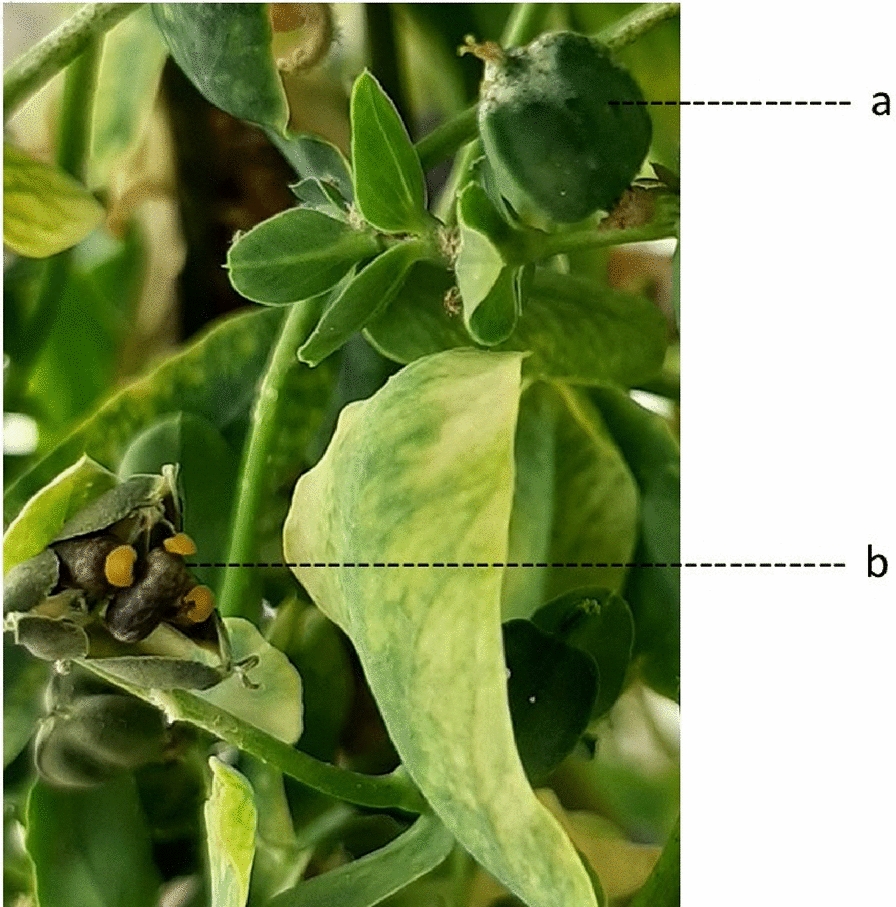
Capsules: **a** non-shattering phenotype of the hybrid plant, and **b** different type of shattering (seeds remain in capsule) for the hybrid plant

**Fig. 12:**
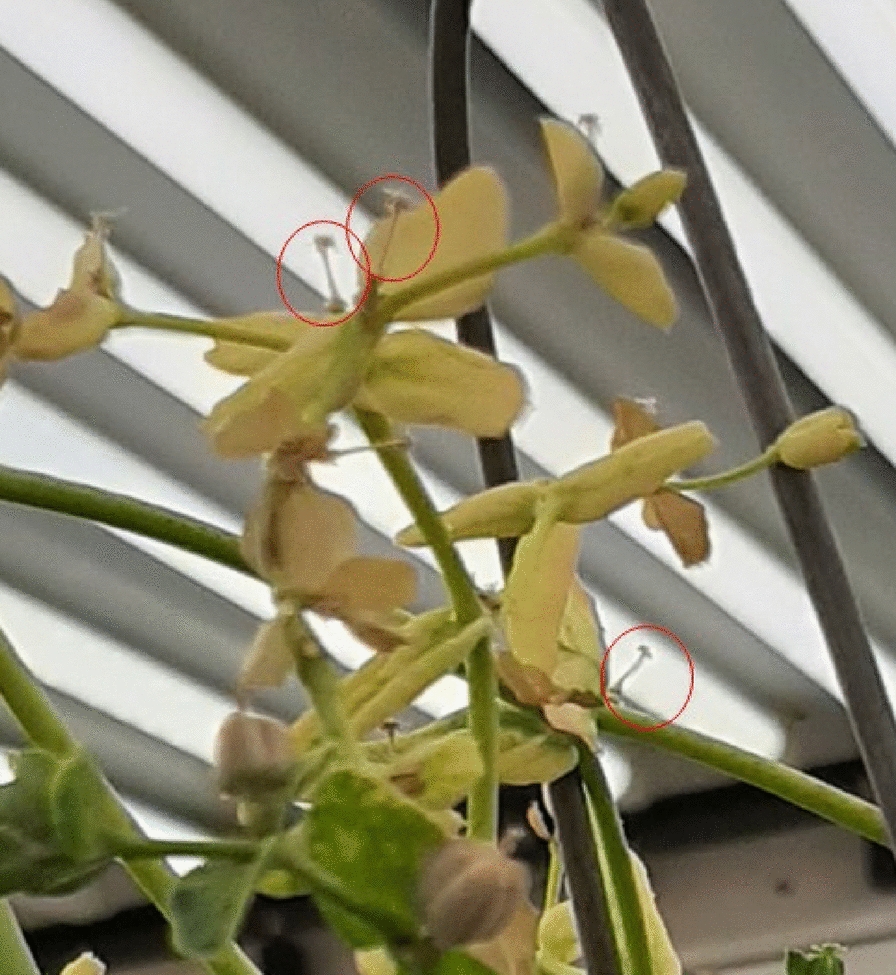
Shattering phenotype for the capsules of the wild-type plants. The capsules are circled in red

Our study indicated that the growing season averages four to 6 months to produce mature seeds, depending on whether the plants are grown outside or inside. When grown indoors, the plants started flowering 2 months after sowing from the top continuing up as one inflorescence included as many as nine flowers per branch. The ability to perform successful crosses is dependent on a thorough understanding of flowering anatomy and behaviour. Observations have shown that female flowers develop before male flowers with flowering beginning in the center of the cyathium. The stigma is receptive and grows to be above the male flowers before the male flowers become fully developed. The pollen matures later than the development of the female flowers which prevents self-pollination. At a point in the reproductive development of this species the cyathium shifts from being dominated by female flower parts to male flower parts. This occurs when the pistillate parts within the cyathium bend and the female flower is aborted leaving only the male flowers. This is a final prevention of self-fertilization in the absence of human intervention. Instead, cross-pollination is enhanced by the presence of insects and wind. In this study we observed that the cyathium consisted of both single female and several male flowers in one structure that started to bloom from the top of the plant (Fig. 9). In the current study, the F_1_ generation from crosses between the wild-type genotypes and mutant genotype produced plants that in turn produced thousands of non-shattering capsules for more than 3 months (Fig. 10). Different types of shattering appeared in the F_1_ hybrid plants at the very late stage of growth, which may indicate a possible simple inheritance of seed shattering, which were investigated in a separate study (data not shown). The non-shattering phenotypes are genetically inherited where the mesocarp is absent as a possible cause of the presence of indehiscent behaviour (perhaps due to an interaction between the growth stage and growing conditions). However, the growing conditions may play a role in inhibiting or enhancing the shattering behaviour that leads to seeds being maintained in the opened capsules (Fig. 11). Regular capsule shattering as appears in the WT plants, including mesocarp in the wall of the capsules is depicted in Fig. 12.

## Discussion

*Euphorbia lagascae* can grow to a height of one meter, with the bulk of seed production occurring in the top third of the plant [4]. This species also produces poisonous milky white latex (Fig. 1), which may cause skin irritation in some cases. In addition, the stickiness of the latex complicates mechanical harvesting if the plants are not sufficiently dry prior to combining [4, 5, 17]. However, the latex may have both pharmaceutical and petrochemical replacement value similar to latex from other members of the subfamily Euphorbioideae [17]. In general, developing an efficient way to cross and breed *E. lagascae* required the study of its botanical structures including leaves, stems, roots, flowers, seeds, and fruits (capsules).

**Fig. 13:**
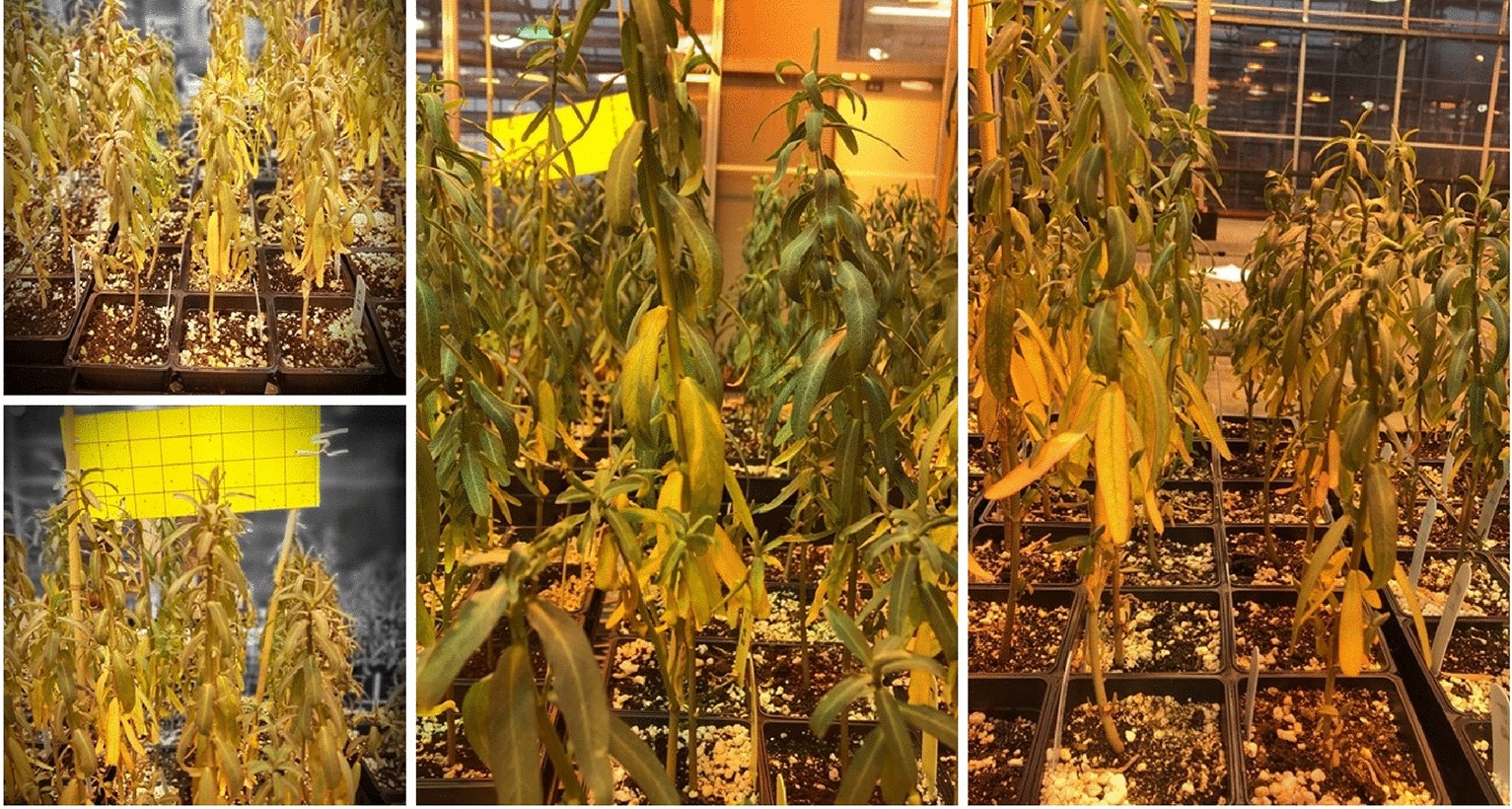
Variations in leaf colour among cultivated lines ranged from yellow to dark green

The results of this study provide a detailed insight into the morphological and reproductive characteristics of *E. lagascae*, which is necessary to initiate a breeding program including efficient hybridization for the crop. In our study, we observed different colour in leaves at different growth stages. S Chakraborty, J Todd, T Isbell and R Van Acker [14] indicated that *E. lagascae* Spreng seed yield, vernolic acid content and oil yield did not increase in response to increasing levels of nitrogen fertilization. Nevertheless, in this study, leaf colour may have been related to the amount of supplied nitrogen, where yellow leaves may have indicated nitrogen deficiency. Moving the plants to a greenhouse with 30% lower relative humidity comparing to the one described by S Chakraborty, S Cici, J Todd, C Loucks and R Van Acker [13] resulted in the producing healthier plants with green leaves (Fig. 13).

The stem height of *E. lagascae* Spreng plants ranged from 20 to 80 cm and displayed either a vertical or horizontal phenotype. The mutant genotype EU006 produced thin, weak stems that grew irregularly even when sown at the same time. S Chakraborty, J Todd, T Isbell and R Van Acker [14] reported that plants grown in Ontario had more branches in 2016, when the weather was hot and dry. The same study indicated that plants grown in 2014 and 2015 under wetter growing conditions displayed enhanced vegetative growth. However, the continuous vegetative growth could also be caused by the different genotypes being grown as a crossing parent. Therefore, in the interest of performing ideal crosses, researchers should take into consideration that the mutant plants take more time for vegetative growth than the WT ones. In contrast, the WT plants with higher germination potential tended to start flowering about a month earlier. As such, to facilitate crossing of the WT and mutant genotypes, the mutant genotype should be planted approximately a month earlier than the WT plants, using staggered planting. If the flowers are available for both genotypes at the same time, performing a successful cross is possible when the pollen is shed, and the stigma is receptive.

*Euphorbia lagascae* propagates by means of both seeds and roots [18]. Using root cuttings, A Ibáñez-Torres [18] reported the possibility of vegetative propagation for *E. lagascae* which could be considered. However, this was beyond the scope and was not explored in the current study. *E. lagascae* has been reported to have a life cycle as long as 8 months [19] when sown in the autumn, assuming that the best agronomic practices included irrigation under warm conditions when the crop is sown in the spring. This same study [19] indicated the importance of breeding for non-shattering phenotypes.

In this study, the F_1_ generation from crosses between the wild-type genotypes and mutant genotype successfully produced plants that produced thousands of non-shattering capsules for more than 3 months. The results of a genetic study for seed shattering and germination in which these F_1_ plants were used are reported elsewhere (Istaitieh et al., under revision). Regular capsule shattering was observed in the WT plants, including mesocarp in the wall of the capsules. A method to grow *E. lagasce* plants indoors to produce successful F_1_ seeds has been described in detail. This in turn allows for a better understanding of the seed shattering trait, which has been pursued using F_2_ generation in a companion study (Istaitieh et al.; manuscript under review). Both the hybridization and the segregation study are critical to fully characterize the genetic control of such an important trait for the domestication and breeding of *E. lagasce*.

## Conclusion

In this study, we have characterized the flower morphology, plant, and capsule development in *E. lagascae*. One of the most important observations in this research is the new shattering phenotype (partially indehiscent capsule) found in the hybrid derived from crosses between the WT and mutant genotypes confirming previous reports. This finding may support the hypothesis of a simple, major gene inheritance with dominance for the non-shattering capsules, which has been addressed in a related genetic study. The environmental conditions such as temperature, moisture and the length of vegetative period had a great influence on how the capsules developed. Accordingly, the capsule shattering behaviour has been separated into two types: (1) genetically controlled with a capsule wall of three layers, and (2) associated with environmental stresses, which warrants further investigation. However, it has been noted that seed retention (i.e., indehiscent behaviour) increased substantially with a 10 **℃** rise in the temperature, regardless of the associated stem lodging. Our findings report the development of a method of efficient hybridization between *E. lagasce* flowers for breeding and genetic improvement purposes leading to further industrial crop development.

## Methods

### Wild-type (WTs) genotypes

Seeds of five wild-type (WT) genotypes (Ames 22,903, Ames 22,906, PI 607,972, PI 607,975 and PI 649,765) originally collected from wild populations growing in different areas of Spain were obtained from the U.S National Plant Germplasm System (NPGS) gene bank. The general characteristic of these five wild-type selections was severe seed shattering making practical seed harvest from these WTs very difficult. The WTs were also chosen to create genetic diversity and increase germination potential in the progeny genotypes that would be obtained from crossing the non-shattering mutant genotypes originally generated by MJP Villalobos and G Robbelen [11].

### Mutant (M) genotypes

Both wild type and mutant genotypes have been used in this study. Many crops display a non-shattering genotype after recurrent selection of spontaneously mutated phenotypes, which is associated with plant domestication [2]. The first commercial mutant genotypes for *E. lagascae* Spreng were registered in Spain [5]. The original mutant seeds (EU005, EU006, EU008) that display the non-shattering phenotype were supplied by Dr. Rich Roseburg from Oregon State University. The first study of these indehiscent (non-shattering) mutants genotypes, produced through ethyl methane sulfonate (EMS) mutagenesis were done by F Verdolini, A Anconetani, D Laureti and M Pascual-Villalobos [12]. In our field studies at Simcoe, the three indehiscent genotypes (EU005, EU006 and EU008) performed well under field conditions when grown from transplant but showed irregular and low rates of germination when directly seeded in the field. The mutant genotype at the greenhouse used for this study was EU006 as a genotype that contributes the non-shattering trait to the progeny. Due to resource limitations, we could only use one mutant, EU006, for crossing with the WT genotypes.

### **Planting genotypes for monitoring flowering behaviour**

Seeds of the wild-type genotypes were planted under controlled greenhouse conditions in May 2018. The growing conditions used were described by S Chakraborty, S Cici, J Todd, C Loucks and R Van Acker [13]: Photoperiod of 16 h (16 h of light, 8 h of darkness), 70% relative humidity, light levels of 350 µEm^−2^ s^−1^, day and night temperatures of 25 and 20 **℃**, respectively. Seedlings were initially grown in one L pots and then transplanted to larger, 4.5 L containers. The pots contained a soil mix of Sungrow, LA4, perlite and sand at a ratio of 5:10:1:1. Accordingly, the amount of perlite and sand facilitate drainage and allows ventilation to minimize root rot. During the 1st month, parental lines were grown without fertilizer and then they were fertilized with 1 kg NPK (20: 20: 20) per gallon with injector of 1:20 ratio; two times per week. The five WTs were used as a female parent because they had fewer male flowers making the emasculation process easier. Concurrently, seeds from the mutant genotype (EU006) collected from field grown plants in 2017 were planted into similar pots with the same soil mix and under the same growing conditions as for the WTs. However, the WTs parental lines and the mutant genotype placed into separate growth chambers to prevent cross-pollination between the different genotypes. The plants were transplanted to different pot types and sizes at various growth stages to allow for the plants to continue to develop and grow. During the study, the fungicide *azoxystrobin* (Quadris; Syngenta) was applied to all genotypes as a protective treatment. Fungus gnats were biologically controlled using the entomopathogenic nematode *Steinernema feltiae* (Nemasys, BASF) and the predatory mite *Stratiolaelaps scimitus*. At flowering time from April to May 2018, female flowers were emasculated using tweezers to facilitate crossing with male plants of the non-shattering (mutant) parental lines. Staggered plantings of different genotypes were done to allow for differences in flowering times between male and female parental lines. Flowering stages were classified as either young or adult. The emasculated female flowers from the WT (young and adult) and the male flowers obtained from the non-shattering mutant genotype (young and adult) were hybridized by collecting pollen from the male parent and putting it on the stigma of the female parent’s flower with a paintbrush manually (Additional file 1: Fig. S1). When crossing, any latex exuded from the plants was removed using paper tissues. Between October and November 2018, capsules from the successful F_1_ crosses were harvested and the F_1_ seeds were stored in the dark at 4^o^C for further research.

### Supplementary Information

**Additional file 1: Fig. S1.** Images of male and female flowers from left to right: both, male and female only.

## Data Availability

Most data are included in the manuscript and supplementary materials. Any additional data and information is available from authors by contacting the corresponding author, Istvan Rajcan, at: irajcan@uoguelph.ca.
