# Supplementary material for: Improvement of germination rate and hybridization to facilitate breeding of an industrial oil crop, Euphorbia lagascae Spreng
Source: Plant Methods. 2024 Jan 24;20:14. doi: 10.1186/s13007-024-01141-2 (PMC10809589; doi:10.1186/s13007-024-01141-2)
Supplement: Supplementary file 1 — Additional file 1: Fig. S1. Images of male and female flowers from left to right: both, male and female only. [file 13007_2024_1141_MOESM1_ESM.docx]

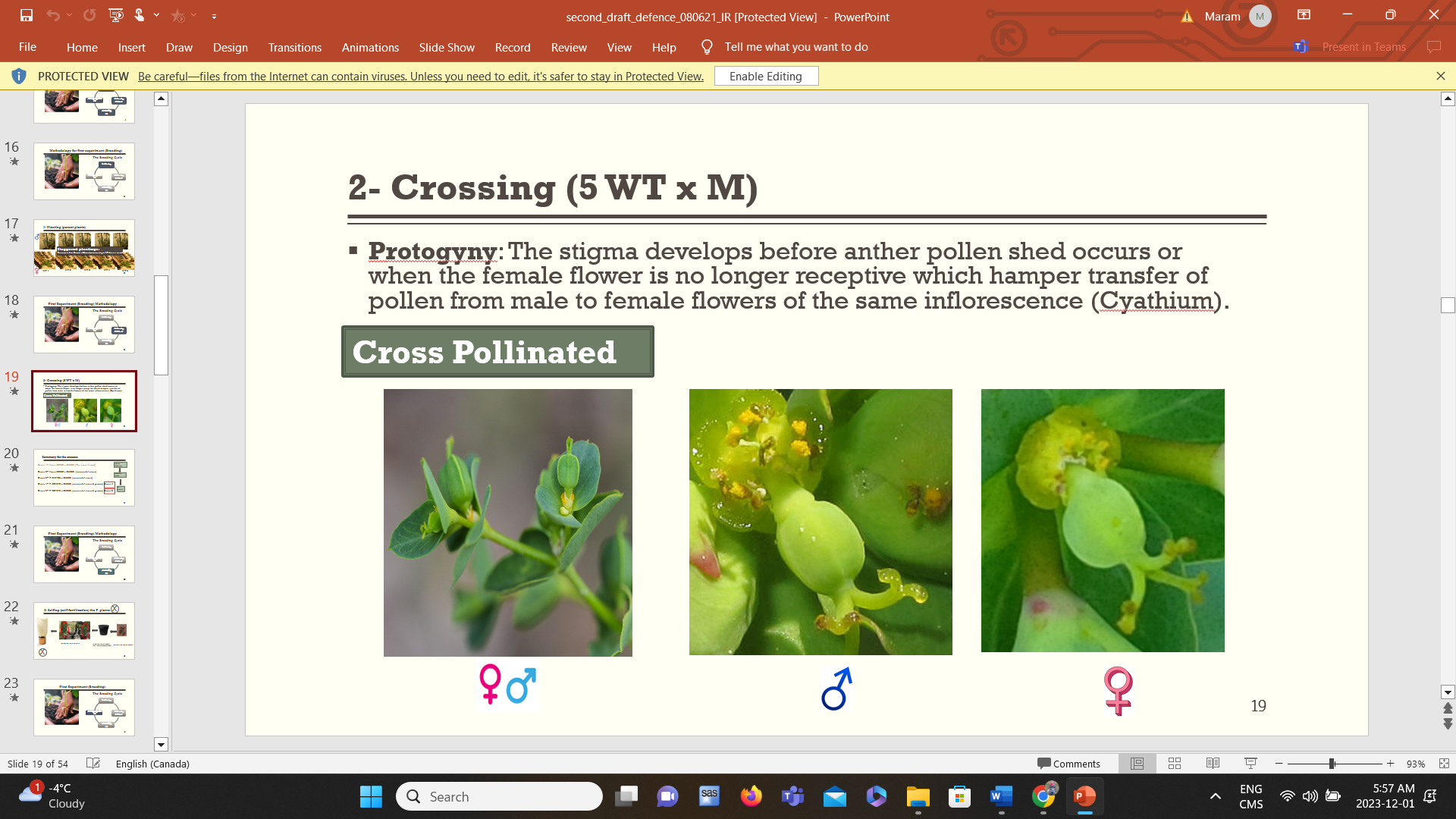


**Supplementary Fig. S1**. Images of male and female flowers from left to right: both, male and female only.
